# Information sharing across generations and environments (InfoSAGE): study design and methodology protocol

**DOI:** 10.1186/s12911-018-0697-4

**Published:** 2018-11-20

**Authors:** Yuri Quintana, Bradley Crotty, Darren Fahy, Lewis Lipsitz, Roger B. Davis, Charles Safran

**Affiliations:** 1 Division of Clinical Informatics, Beth Israel Deaconess Medical Center, 330 Brookline Avenue, Boston, MA 02115 USA; 2 Harvard Medical School, 25 Shattuck Street, Boston, MA 02115 USA; 3 Medical College of Wisconsin, 8701 W Watertown Plank Rd, Milwaukee, WI 53226 USA; 4 Froedtert Hospital, 9200 W Wisconsin Ave, Milwaukee, WI 53226 USA; 5 Hebrew Senior Life, 1200 Centre St, Roslindale, MA 02131 USA

**Keywords:** InfoSAGE, Internet, Information and communication technology, Eldercare, Informal caregiving, Shared decision making

## Abstract

**Background:**

Longevity creates increasing care needs for healthcare providers and family caregivers. Increasingly, the burden of care falls to one primary caregiver, increasing stress and reducing health outcomes. Additionally, little has been published on adults’, over the age of 75, preferences in the development of health information sharing with family members using online platforms. This study aims to assess a novel, Internet based, family-centric communication and collaboration platform created to address the information needs of elders and their informal caregivers in a community setting.

**Methods:**

This study is an internet-based, open prospective cohort study, enrolling dyad pairs of one adult over the age of 75 with one informal caregiver. Dyads will be offered to use the InfoSAGE online platform without prospective assignment. Participants will consent using an online process that enables participation from any location and shares important study and privacy details. The platform will enable the capture of search queries and tracking of functions such as tasks and discussions. Surveys every six months assess health status, health and social needs, and caregiver burden using validated instruments over a two-year period. We will use a mixed methods approach, utilizing qualitative survey data along with website usage analytic data.

**Discussion:**

Analysis of the longitudinal usage and survey data will help to examine the patterns of family communication and health information seeking as the central older adult ages. We will use the study data to inform design recommendations relevant to a complex mixture of users, with special consideration to the needs of older adult users and potential physical limitations.

## Background

Recent census information indicates that the population over the age of 65 is increasing at a faster rate than any other age group, and the population over the age of 85 is expected to double by 2040 [1]. Families will likely need to play an increasingly important role in the caretaking and well being of the elderly. Greenberger [2], Cho [3], and others, for example, have emphasized the increasingly important health-‘facilitating’ role that family members assume. This ‘facilitating’ role includes such things as helping to maintain independence and autonomy, administering care, directing the elder to healthy behaviors and providing health-related information. As important, the role also involves “positively manipulating the environment, recruiting other individuals to assist, negotiating [healthcare system] bureaucracy, and optimally rearranging the care-recipient’s living accommodations” [2].

Even with the increasing need for familial support, each year a larger proportion of individuals will live alone (spouseless), and at a distance from immediate family members [4]. More often than not, neither the elder nor their family how to readily access or share health information [5].

As described by Agarwal and Khuntia, consumer health information technologies (IT) could play a role in reducing this vulnerability [6]. In order to successfully do so for this unique population, special considerations need to be given to the design and functionality of these tools and resources. First, the concept of the ‘user’ must be flexible, and the underlying design of the technology must be capable of accounting for a variety of ‘user’ models. In some cases, the ‘user’ will be the independent elder, whose physical capabilities can diminish over time. In other cases, the ‘user’ may be a network of elder and family caregivers. In still other cases, the ‘user’ may be a designated healthcare proxy.

Second, there is a need to increase our understanding of the information requirements, information management practices, preferences, and priorities for any of these ‘user’ models – a topic about which very little has been published.

The current understanding of how the independent elder seeks and uses healthcare information is limited. Some studies have examined elder use of information technology and the Internet to support their health information needs. Campbell, et al. [7, 8], for example, found that with training, elders were both willing and successful in using the Internet to find relevant health information. However, as a group, these elders perceived the value of the information retrieved to be low, and they continued to prefer direct contact with a traditional healthcare provider as their primary source of personal health information.

A study by Leaffer and Gonda reported that Internet use by an elderly population to meet their health information needs appeared to be more sustained [9], and a significant number of subjects reported an increase in satisfaction with their medical treatment during the period of Internet use. The investigators observed, moreover, that in this elderly population, women seem to be more motivated than men in searching for health information on the web.

The major limitation of these and other studies, however, is that they have not examined, in detail, how the information needs of the elder evolve over time, how information acquired from consumer sources shapes decision-making, and how needs and behaviors change in response to specific health events [10–14]. Additionally, these studies under-represented the “oldest old age group.” In the Kaiser Foundation report, for example, only 9% of the respondents were age over 75 [15]. Finally, these studies do not differentiate between the needs of a fully independent elder and one who has chosen to or needs to share governance over personal health information with an extended family [15].

With respect to the information management practices, preferences, and priorities for the *informal caregiver or proxy*, our understanding also is patchy. A number of studies have identified that for a family member who is engaged in day-to-day care for an elder, access to healthcare related information can be an important mediator of stress, and can measurably influence the effectiveness of the caregiver. Few studies, however, have elaborated on specific types of information, specific use cases or other detailed requirements. For example, Bakas, et al found that caregivers of stroke victims for whom they were providing long-term in-home care consistently expressed a need for better access to information [16], as well as informal social outreach, but the study did not elaborate on how and in what specific contexts this information might be most useful. Hills documented that lack of access to therapeutic goals (as would be provided by a formal caregiver, such as the primary care physician) contributed to frustration among informal family caregivers [17]. A 2002 cross-sectional study conducted by Greenberger and Litwin confirmed that access to health-related informational resources represented a key element of support required of family members who were helping to care for dependent elderly [2], but again did not provide detailed implementation guidelines. Finally, Buckley, et al evaluated a specific health-IT intervention [18], telehealth, and found that the technology was embraced by in-home, family caregivers of stroke survivors to seek informational and emotional support not only for the patient, but also for themselves. Clearly, when considering the features and functionality of consumer health IT for the elderly population, one must consider not only the elder but also the informal caregivers within their family.

New technologies provide great opportunities to enhance the quality and safety of healthcare. However, consumer healthcare IT is biased to the young, relatively independent user. It is rare to see underlying designs capable of simultaneously supporting specific physical and cognitive limitations of a user, or more general needs of an elderly population, despite published guidelines relating to readability, presentation of information, ease of navigation and incorporation of other media [19–23]. It is even rarer to see designs that can accommodate evolving models of the user, such as are required when family members begin to share decision-making and management of care with their elderly parents or grandparents.

## Methods/design

In order to evaluate the care communication and collaboration needs of aging elders and their families, we have specified four specific aims for this project: (1) To create a novel, family-centered information management and collaborative environment that is based on the requirements and needs identified through our ongoing research; (2) To identify the information needs and decision-making dynamics of elders and those helping to care for them, with a particular focus on how needs evolve as elders transition from full independence to family-supported care; (3) Using our laboratory developed web-based platform, to longitudinally study patient and family collaborative interactions and information management behaviors in the context of real healthcare decision-making and care tasks; (4) To evaluate the extent to which our platform improves communication, coordination, and collaboration for elders and their family members through surveys.

### Design of the InfoSAGE platform

The InfoSAGE platform enables family members and friends to connect around a particular person (i.e. the keystone) for care and social coordination, with areas for conversation, medication management, and shared task lists [24]. We used focus groups [25, 26], as well as best practices and heuristics drawn from the literature to inform the design of the platform [27].

Multiple privacy rings were designed to control the types of information being shared. The InfoSAGE platform was designed in such a way that independently functioning older adults could control the network and privacy settings, but that a designated proxy could also control all areas should the keystone delegate access. In the case that the keystone would or could not engage in the platform, family members could create accounts on the keystone’s behalf using the proxy function as well. If keystone participants would later want to become users, they could then activate their account.

The platform is available free of charge for families on the web (https://www.infosagehealth.org) and available as a mobile application for Apple’s iOS and Google’s Android operating systems.

### Participants, recruitment, and setting

To answer the questions set forth in the specific aims, we will employ a prospective observational cohort of patients over the age of 75 and their family members. While any person/family is able to join and use the service, we will invite families at the time of sign up to participate in an open, prospective cohort study. Users can self-select into the study at the time of account creation if they meet eligibility criteria and are willing to share data about their use of the platform with the research team, as well as to respond to biannual surveys delivered electronically through REDCap [28]. We plan to recruit up to 250 families to participate in this evaluation.

#### Eligibility criteria

Given our initial focus on the information needs across a diverse group of aging individuals and their families, we will have relatively few exclusion criteria. Dyads will be enrolled based on the following eligibility criteria. The person at the center of the care network, also known as the keystone participant, is an older adult defined as age 75 or greater as of the time of enrollment. These elders will be community dwelling, meaning that they live in a private residence, a continuing care retirement community, subsidized senior housing, or assisted-living. We will not include those seniors who live in skilled nursing facilities permanently. As the study will focus on caregiving, the keystone or index senior must have a family member who is willing to participate in this project. Family members, who care for an eligible individual, may also join the study if their keystone relative is not able to participate in the project, but meets other inclusion criteria, such as age and residence location. Participants providing data must also be native English speaking given that the InfoSAGE platform and survey questionnaires are available only in English. The family caregivers must be involved in the Keystone elders’ life and care, though not necessarily local to the area. Participants may have personal access to the Internet or be able to share common access to the Internet at a public or housing facility. Participants will be based in the United States.

During our recruitment, we will have relatively few exclusionary criteria. As such we are likely to enroll a number of subjects who have moderately diminished cognitive capabilities (potentially undiagnosed). We acknowledge that there may be some instances where an ‘index elder’ is enrolled, but due to memory or other cognitive deficits does not become an ‘active’ user of the system. In these cases, however, there may still be active use of the system by the designated network of family caregivers, generating evaluable data.

We will recruit participants through invitations to residents of collaborating senior housing facilities, including mailings. We will partner with primary care and geriatric offices and social workers as well to advertise the availability of the website and the ability to participate in the research study.

### Ethical and privacy considerations

**Fig. 1:**
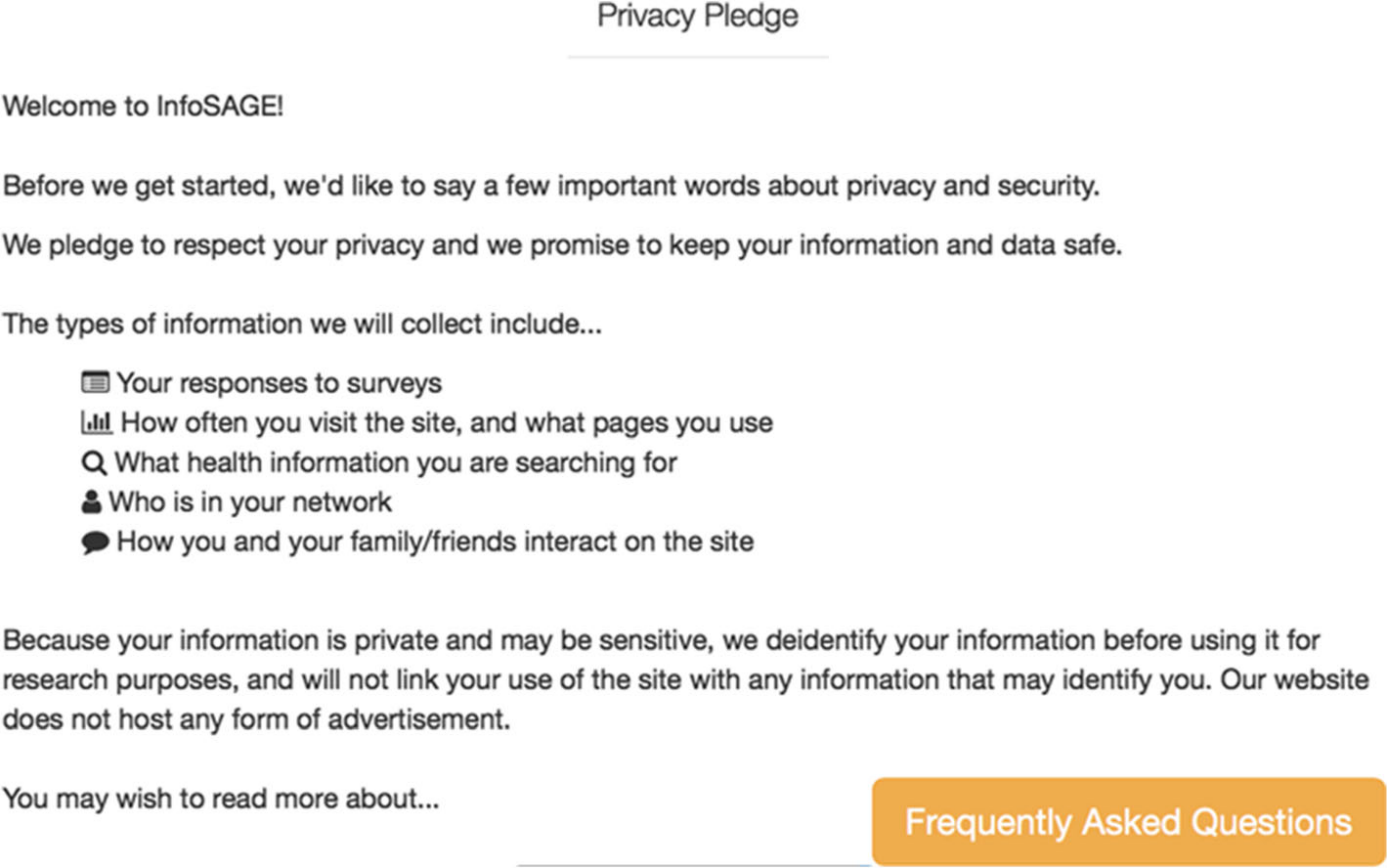
User privacy pledge. Upon registration, users are prompted to review and acknowledge the types of information collected by the InfoSAGE platform, and may select to review frequently asked questions

**Fig. 2:**
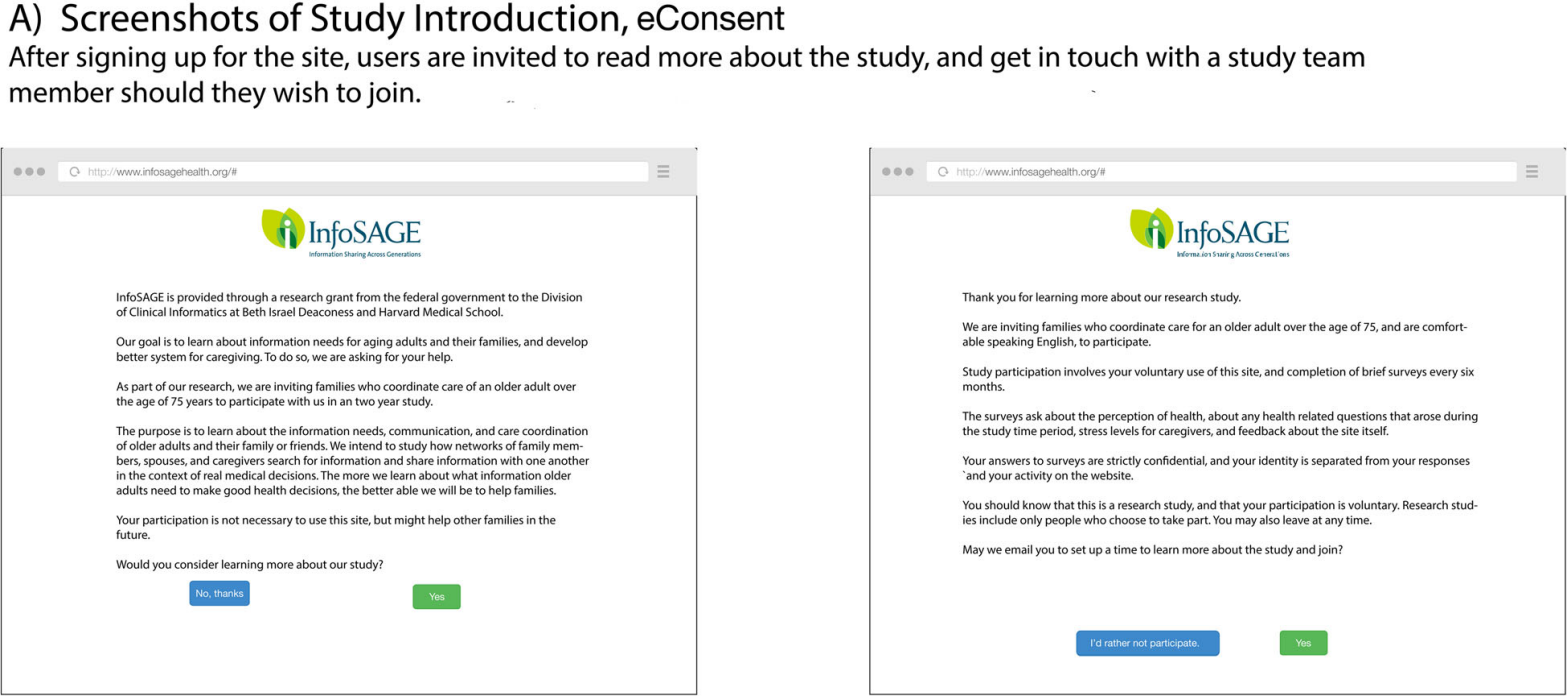
eConsent screen. Sample eConsent screen displaying the consent process. The first screen introduces the study in plain language. The second screen describes the study in more detail and allows the user to opt into the research cohort

The protocol received approval by the Institutional Review Board of Beth Israel Deaconess Medical Center. Information about the research study will be provided using an online process as part of the new user registration process (Fig. 1). A modification of the informed consent process was created to provide participants with plain-language information about the study and how data will be used for research purposes (Fig. 2). To carry out the research aims, we will link data together, including survey data, site activity, and basic demographics. Research data will use a unique study id.

The online InfoSAGE application has multiple security and privacy layers. The application will be only accessible only via RSA-certified Secure Socket Layer based browser connections. This will allow a highly secure and reliable connection, assuring that information cannot be intercepted en-route to and from the servers. Data between sites will be sent fully encrypted using AES- 128 (Advanced Encryption Standard), meeting NIST standards.

### Data collection

**Fig. 3:**
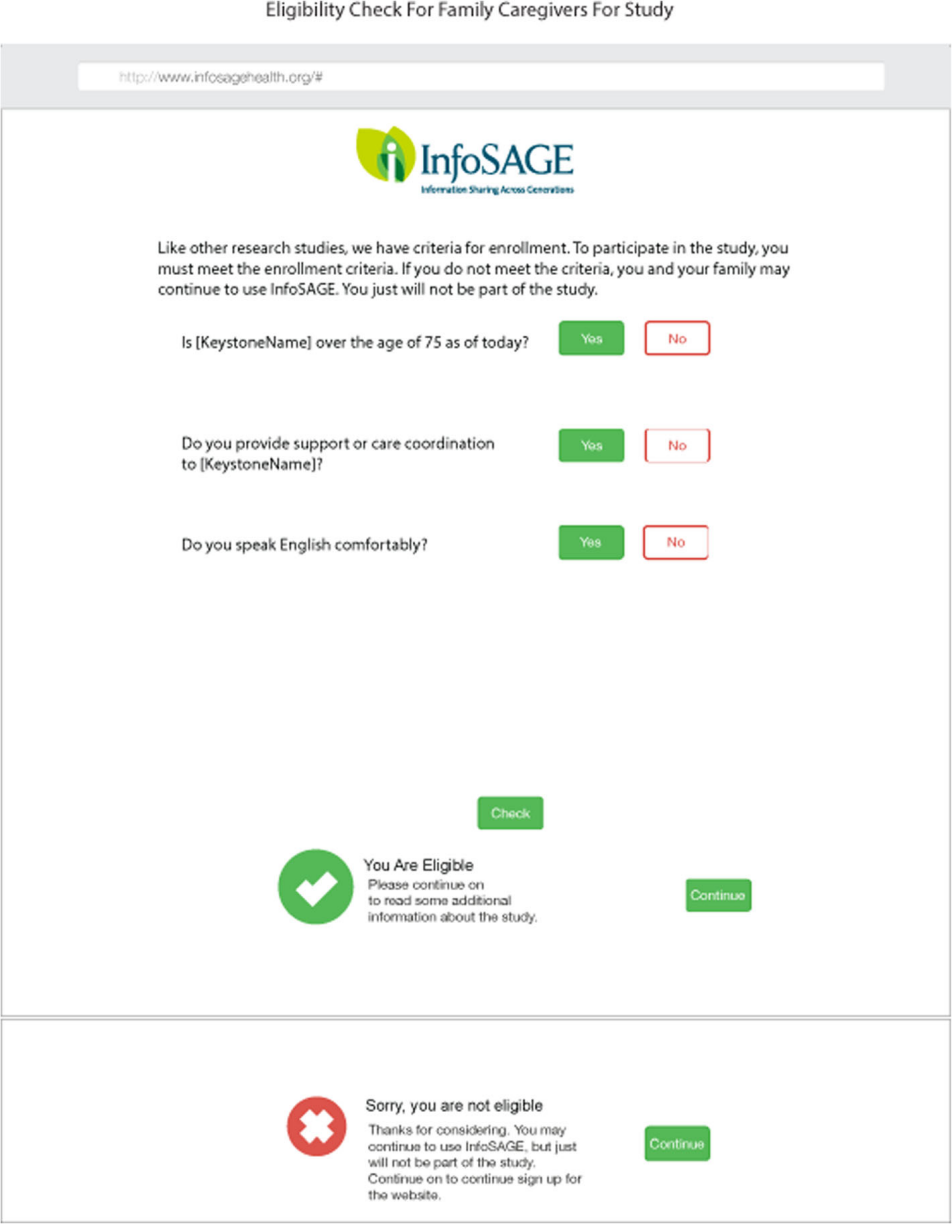
Eligibility verification. Review of eligibility requirements. A response in the negative to any of the eligibility questions disqualifies the user from study participation

**Fig. 4:**
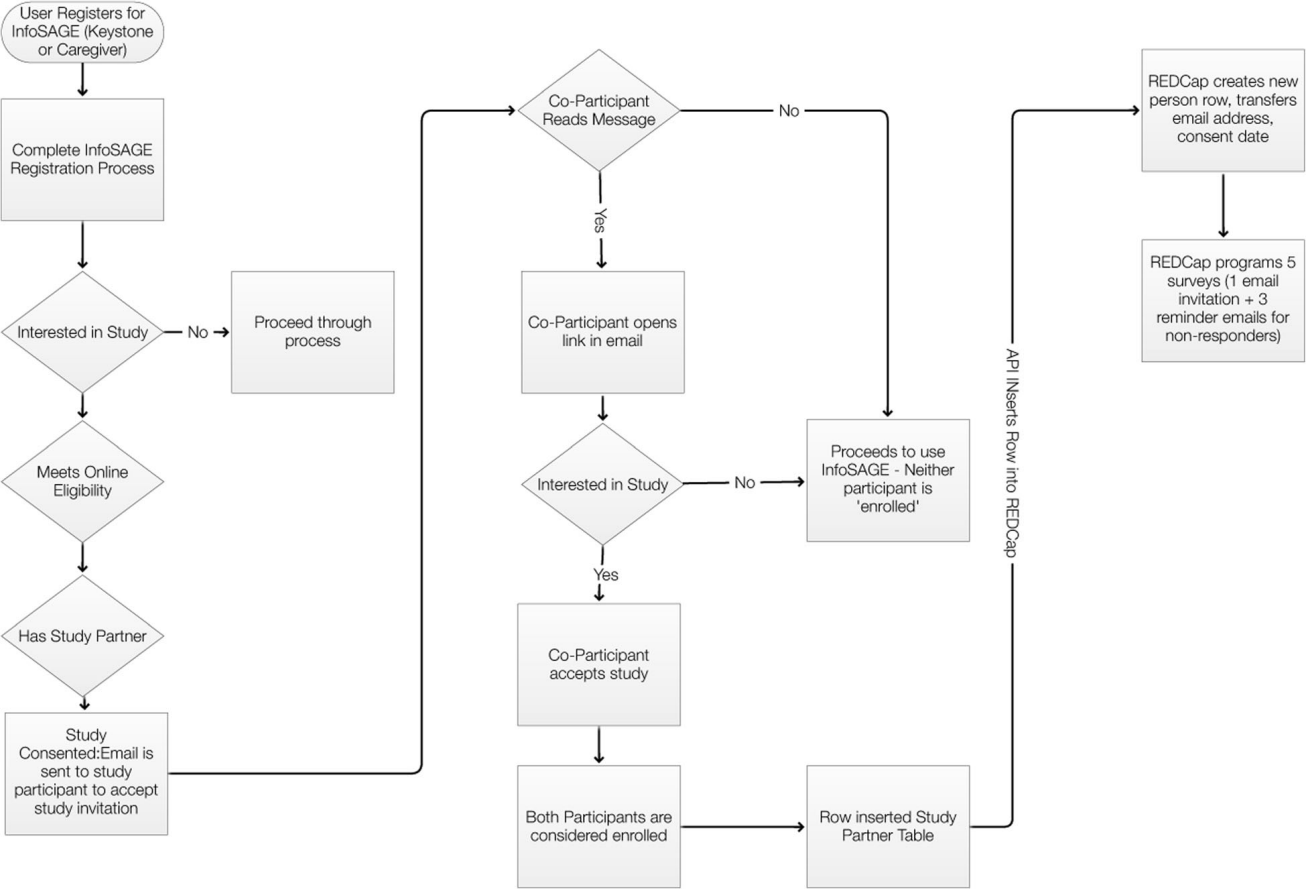
Registration, enrollment, and data collection flowchart. Overview of user registration, enrollment, and data collection through REDCap. Surveys will be automated through the website and recorded in REDCap

At the time of website enrollment, users who meet the inclusion criteria and none of the exclusion criteria will be asked to opt into the cohort/study. Users will read information about the study and what it means to “opt in” on the website, and complete a short form to verify eligibility (Fig. 3). User registration and data collection is outlined in Fig. 4.

**Fig. 5:**
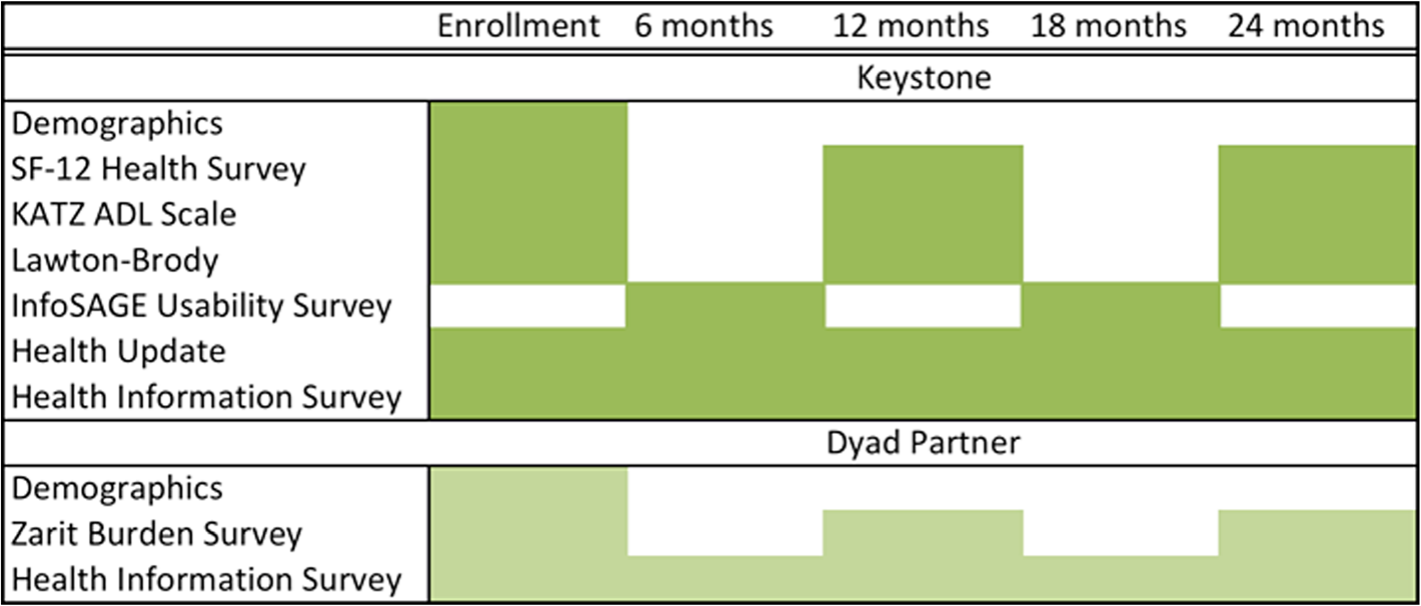
Schedule of surveys and instruments

**Fig. 6:**
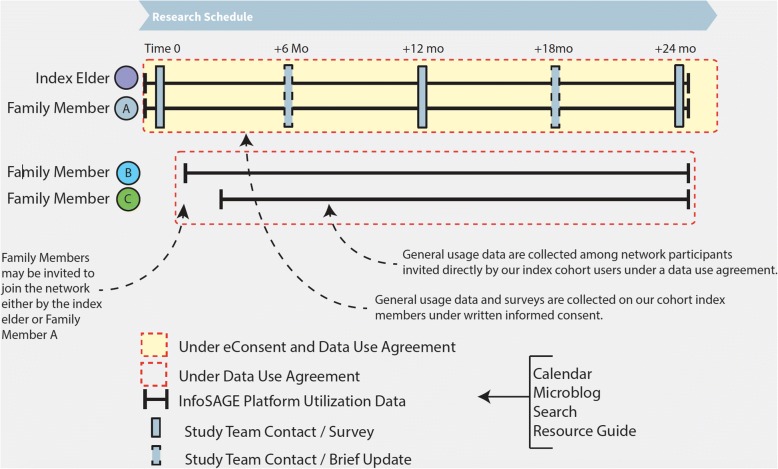
Longitudinal research schedule

All study participants will be asked to fill out a study questionnaire/survey at the time of enrollment. This will be delivered through the Internet using REDCap. Participants will be invited to use our information platform and we plan to be in touch with them every 6 months for brief telephone surveys, and every 12 months for a longer follow up survey (Fig. 5). We will contact them by phone or email, and/or regular mail to remind them of scheduled questionnaires unless they have opted out of the study. We plan to follow this cohort for 2 years (Fig. 6).

Through our platform, we also plan to assess what resources patients and family members actually search for using our search functionality and resource guide.

This involves prospective data collection of search queries and browsing data for the resource guide. This will help us understand how seniors and family members formulate their information searches (syntax and complexity), general content areas (clinical information, disease specific, aging specific, logistic information, housing, durable medical equipment), and utility.

Participants in the study opt into having their searches analyzed, though we will protect the security of their identities. Where possible, we will de-identify any of the search queries and browsing habits. We will retain some linking to develop understanding of their context, such as where they are living (at home, at a retirement community, or assisted living), and their health trajectory.

**Fig. 7:**
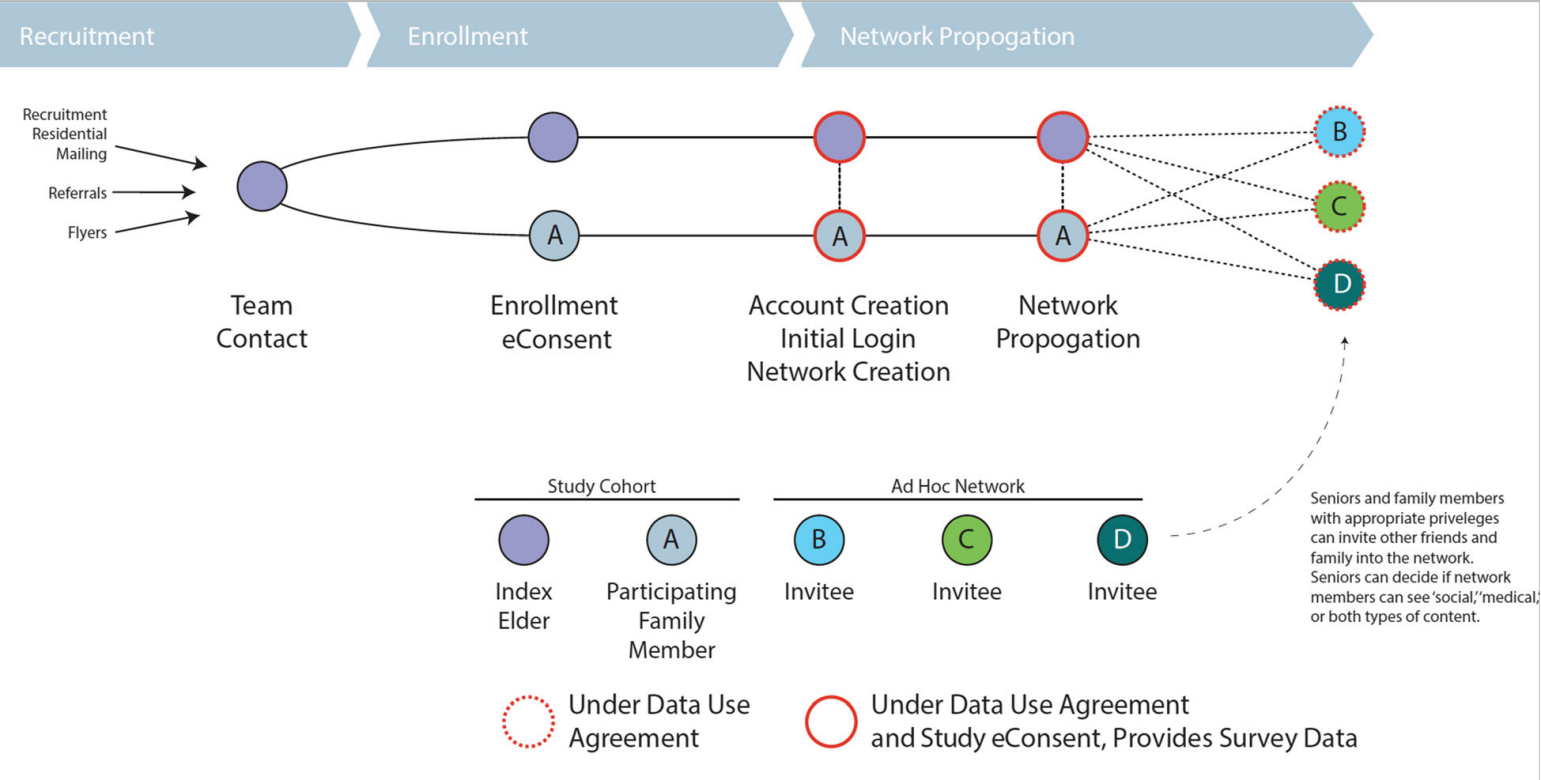
Network propagation and data use

We will not formally enroll other family members, though our user agreement for the platform notifies users of our academic mission and our use of data to help inform our elder network analysis and information needs (Fig. 7). The study team will have no direct contact with these family users.

### Data analysis

Patient, family and provider assessments of communication, coordination, collaboration, and care are measured 3 times (baseline, year 1 and year 2). We will treat the outcome measures as continuous variables and fit linear mixed effects regression models treating patient as a random effect. Independent variables in the primary models will be treatment group, time and a time-treatment interaction. The focus will be on the Wald test for the interaction. As sensitivity analyses, we will fit additional models that adjust for variables that are found to be associated with the outcome measures (e.g., age, sex, functional status, medical conditions, etc.). To better understand our data, we will also conduct a variety of exploratory analyses. We will identify correlates of the outcome measures. We will also examine correlations between patient and family assessments and between patient and provider assessments.

Information queries will be analyzed descriptively. In order to longitudinally analyze health information utilization in the InfoSAGE, we will use a combination of strategies that are collectively referred to as process mining. The process mining analytic tools will enable us to perform the following high-level analytic tasks: (1) Analyze information sharing and interaction between patient-family-providers in the collaborative environment around specific tasks, and in relation to specific events. Examples of representative events include ambulatory office visits or discharges after acute inpatient hospitalizations (assessed by responses on the health update survey); (2) Analyze the specific type of information that is exchanged within the network after a specific event, or in conjunction with specific tasks. Examples of information include resources retrieved from Internet searches, calendar entries, task entries, and microblog entries.

We will assess usability, satisfaction, and caregiver burnout, and assess the impact of our InfoSAGE platform on the above outcomes. We will survey both the keystone elders and the family members. We will conduct our analysis for these outcomes with predictors including utilization of the platform, utilization of the search functionality, utilization of the peer coordination component such as calendar, task list, and microblog, and size and shape of the network. We will adjust for baseline quality of life, baseline functional status, healthcare service utilization including inpatient or observational studies, rehabilitation stays, and major health events such as new or worsening diagnoses.

### Limitations

This is an open internet-based cohort study, and subject to participant selection bias. It is being deployed in the community, rather than at any single health care organization, which may delay recruitment or bias towards people with less acute needs. The basic approach to analysis of the trial involves the use of longitudinal repeated measures methods. We recognize that we are considering multiple outcome measures in the study, but since this is a technology evaluation/assessment study, we will not explicitly adjust for multiple comparisons. We will acknowledge this limitation and advise appropriate caution in interpretation when we publish our findings.

## Discussion

We are deploying a family-centric information and networking tool, developed in our laboratory, and conducting a preliminary assessment of its utility. We plan to follow participants who are interested in contributing more to the research on aging and care coordination through a prospective cohort study. This will provide information on the nature of information queries, communication domains, and care coordination needs in an aging population.

Our study design is ideally suited for online recruitment, using an online consent process, which has been sparsely applied in the current literature. This relatively novel approach to study recruitment is associated with unique benefits and challenges. As the website is available throughout the United States and the world, recruitment will not be geographically limited to the study facilities, and the study scales appropriately without the need for additional study coordinators. In addition, self-enrollment provides flexibility to potential participants to review study materials and goals prior to joining without the need to schedule time with researchers.

Conversely, we recognize that an online enrollment and eConsent process introduces research and logistical challenges. Potential participants will provide demographic and baseline information which may be difficult to verify, presenting data integrity obstacles. Furthermore, survey responses may be inconsistent and response rates diminished when compared to a mail, phone, or in-person methods of surveys used in traditional qualitative research. Our team plans to verify the veracity of information and data supplied by subjects recruited online, and we will require two or more members’ assent before disqualifying suspect data.

The use of such a recruitment method also has the potential to be self-selecting for technological familiarity. Inherently, if the platform is discovered organically through Internet searches or social media, a certain familiarity and comfort with Internet based technologies is implied. This could create a barrier for wider applicability of the work.

Lastly, an open enrollment will potentially make it more difficult to determine if a user belongs to a collaborating community if the participant does not indicate it, creating difficulties in tracking community based goals. We also recognize that there may be further unanticipated challenges.

If successful, our work will have the following impact: (1) Provide public and private stakeholders with a clear understanding of the role that consumer health IT can play in the complex and distributed care of the aging population; (2) Provide public and private stakeholders with an understanding of how thoughtfully-designed, context sensitive, consumer health IT can measurably change family communication, coordination, and collaboration in the care of elders; (3) Establish a formal framework for assessing the needs, preferences and prevailing behaviors of complex users and successfully mapping them to technical requirements; (4) Establish a formal framework for measuring outcomes related to consumer healthcare IT use; (5) Provide developers of consumer healthcare IT with an accurate and operational model of the ‘users’, use cases, design requirements, functionality and infrastructure required to support the information management needs of elders and their adult children/caretakers. The models will be generalizable to a broad range of consumer healthcare IT applications and when applied, this information will lead to a better technical capability of these applications.
